# Estimating peak breeding season, size at first maturity and variation in fecundity and egg-size at different sizes of Hilsa (*Tenualosa ilisha*)

**DOI:** 10.1016/j.heliyon.2023.e19420

**Published:** 2023-08-23

**Authors:** Md Sayeed Abu Rayhan, Md Shohanur Rahman, Protick Kumar Bose, Md Golam Sarower, Muhammad Yousuf Ali

**Affiliations:** Fisheries and Marine Resource Technology Discipline, Khulna University, Khulna-9208, Bangladesh

**Keywords:** Hilsa shad, Tenualosa, Breeding season, First maturity, Fecundity, Egg-size, Lobe

## Abstract

Hilsa shad, *Tenualosa ilisha*, has recently gained momentum due to its taste, nutrition and demand. Imposing ban at peak breeding and setting up a minimum capture size are two of the most effective tools for conservation and management of any fish species. Although, Bangladesh government has been imposing ban on a particular time and set the minimum legal size, there is still contradictory information on these two issues. That is why, a study was carried out to determine peak season of breeding and first maturity of Hilsa collected across the natural habitats in Bangladesh. Variation in fecundity and egg-size at different sizes were also investigated. Peak breeding season was identified observing gonadosomatic index of female Hilsa all the year round. First maturity was estimated with Probit analysis.

Based on the gonadosomatic index of gravid females, peak breeding season was identified as late October to early November in this study. However, another minor peak was found in February–March. The highest GSI value (12.1) was observed in October–November, which indicates the peak time of spawning. The size at maturity (M_50_) of female Hilsa was estimated as 31 cm total length. A significant variation was observed in egg sizes between the left and right lobes of the fish (t _*(24)*_ = 2.42, p = 0.02), and between the parts of the same lobe (p = 0.03). However, fish length and weight had no effect on egg size(for egg-size vs. fish length, r = -0.009, p = 0.966; and for egg size vs. body weight, r = 0.132 and p = 0.530). The average egg count from left lobe and right lobe were recorded as 0.375 ± 0.16 million, 0.371 ± 0.17 million. Total fecundity was counted as 0.745 ± 0.33 million. A significant positive correlation was noticed between length, weight, and total fecundity (r = 0.7 for total fecundity vs. body weight; r = 0.6 for of total fecundity vs. length). No significant variation was observed in fecundity between the left and right lobe (p = 0.6) and among three parts of a lobe (p = 0.3). The size of eggs varied between two lobes and among different parts of a lobe. The eggs from middle part were bigger in size than the other parts. The findings of our study will help conserve and manage the natural population of Hilsa (*T. ilisha*) in Bangladesh and contribute to the wider scientific community.

## 1 Introduction

Hilsa shad, *Tenualosa ilisha* is one of the most prominent fish in Bangladesh, India, and Myanmar due to its delicious taste and nutritional benefits [1,2]. Bangladesh alone shares about 86% of the total worldwide Hilsa production [3]. Recently Hilsa fishery has gained a great momentum in Bangladesh which supports the livelihood of millions of people [[4], [5], [6]]. It has recently been acknowledged as a geographical indicator (GI) of Bangladesh. Previous studies show that Hilsa has a major spawning season during September-October [7] or October-November [8] depending on the full moon and new moon. During their primary breeding season, gravid Hilsa are widely collected, especially from the primary spawning sites. To prevent indiscriminate hunting of Hilsa and to safeguard its spawning and nursery sites, the Department of Fisheries, Bangladesh, has been implementing a ban on catching, transporting, and marketing of all sorts of Hilsa during the peak spawning season of Hilsa since 2007. However, breeding season as well as peak spawning time depends on the sight of full moon and varies with the changes of some other climatic factors such as tidal action, floods, and rainfall. Therefore, this may need revision through proper scientific study because there is conflicting information about breeding season of Hilsa in Bangladesh. Some reports from social and print medias, and our personal observation suggest that even after the banning period of Hilsa, a huge number of fully gravid Hilsa are captured from many parts of the country, particularly in the upstream of Padma, Meghna, and Jamuna [9]. Thus, for sustainable development of Hilsa fishery in Bangladesh it is important to precisely determine the breeding season and peak spawning time based on scientific study.

Establishing a legal capture size based on a species' first maturity, is another effective tactic in fisheries conservation and management. In Bangladesh, harvesting of Hilsa below 25 cm is prohibited for 8 months from November 1 to June 30 under the ‘Protection and Conservation of fish Act-1950’ [10]. However, the rule is very general for all types of Hilsa and according to our personal observations and conversations with Hilsa catchers, there is little enforcement of this law. The size might need revision through comprehensive scientific study, since the morphometric and meristic characteristics of fish may vary due to climate changes, hydrological parameters, geographical location and habitat alteration [[11], [12], [13], [14]]. Thus, it proves enormous scope to clearly estimate the size at first maturity of Hilsa with recent data.

For studying reproductive biology and estimation of relative size of eggs and absolute fecundity according to gravimetric method, eggs are taken from the front, middle and rare (back) part of each ovarian lobe of the individual. However, there is no scientific evidence that size and number of eggs in different parts and different lobes vary in practical. We do not know if eggs from different parts of an ovarian lobe and within two individual lobes vary in terms of count and size. Therefore, this is important to determine if considering eggs from different parts of each ovarian lobe is necessary or samples from any a part of any lobes in each gonad would result in the same outcome. Although there are several published reports about Hilsa's artificial breeding [[15], [16], [17]], larval rearing and weaning of fry for the development of fingerlings [18,19], no work focused on inter- and intra-lobe variation in fecundity and egg size of Hilsa. Thus, it is essential to have insights into variation in fecundity and egg-size at different sizes of Hilsa.

Having considered all the above-mentioned issues, the present study aimed at estimating peak breeding season, size at first maturity and observing variation in fecundity and egg-size variation at different sizes and lobes of Hilsa fish collected from across the natural sources of Bangladesh.

## 2 Research methodology

### 2.1 Sample collection

**Fig. 1:**
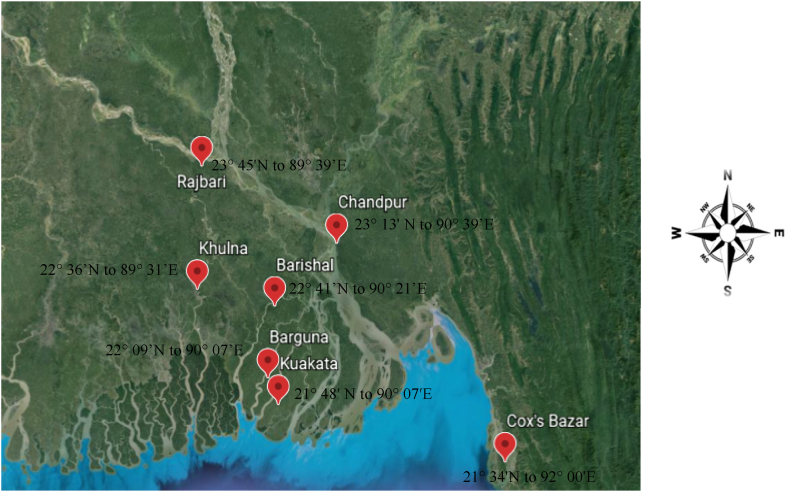
Sampling locations of Hilsa shad across its habitats in Bangladesh.

In order to determine peak breeding season, a total of 155 female Hilsa were collected directly from the river at seven locations of Bangladesh, viz. Rajbari (23° 45′N to 89° 39′E), Chandpur(23° 13′ N to 90° 39′E), Cox's Bazar(21° 34′N to 92° 00′E), Barishal (22° 41′N to 90° 21′E), Barguna (22° 09′N to 90° 07′E), Kuakata (21° 48′ N to 90° 07′E) and Khulna (22° 36′N to 89° 31′E) (Fig. 1 and Table 1). Sampling was carried out from August 2019 to June 2020 once in a month during the full moon directly from the fishers. The data were recorded from the samples of the locations separately. For estimating size at first maturity a pooled sample of 391 were used; and for fecundity and egg-size variation study a total of 25 samples were used for (Table 1). All the samples were stored at −20 °C for analysis in the biology lab of the FMRT Discipline, Khulna University.

**Table 1:** Details of samples used for the analyses.

Location	Sample size	Length cm	Weight g	GSI av.	Comments
Determining peak breeding season					
Kuakata/Months					Samples from Kuakata
January	17	37.7 ± 3	688 ± 194	5.24	”
February	15	34.6 ± 2	581 ± 186	10.69	”
March	08	35.8 ± 6	822 ± 230	10.12	”
April	08	36.5 ± 5	650 ± 283	7.67	”
May	10	38.4 ± 7	827 ± 292	7.42	”
June	10	37.5 ± 4	854 ± 286	5.00	”
July	12	38.4 ± 3	667 ± 246	5.05	”
August	16	38.2 ± 4	756 ± 257	7.93	”
September	14	36.1 ± 3	553 ± 95	8.48	”
October	15	39.3 ± 3	747 ± 128	12.10	”
November	12	34.0 ± 1	747 ± 168	9.59	”
December	18	37.0 ± 4	667 ± 180	6.49	”
Total	155				
Estimating 1st Maturity					-All the samples pooled together -No comparison among the locations
Rajbari	25	–	–	–	
Chandpur	70	–	–	–	
Barishal	27	–	–	–	
Cox's Bazar	35	–	–	–	
Barguna	14	–	–	–	
Kuakata	145	–	–	–	
Khulna	75	–	–	–	
Total	391	33.6 ± 7	549 ± 320		
Fecundity and egg size study					
Kuakata (Jan–Feb)	25	36.2 ± 4	640 ± 219	NA	Samples only from Kuakata

### 2.2 Data recording and image processing

**Fig. 2:**
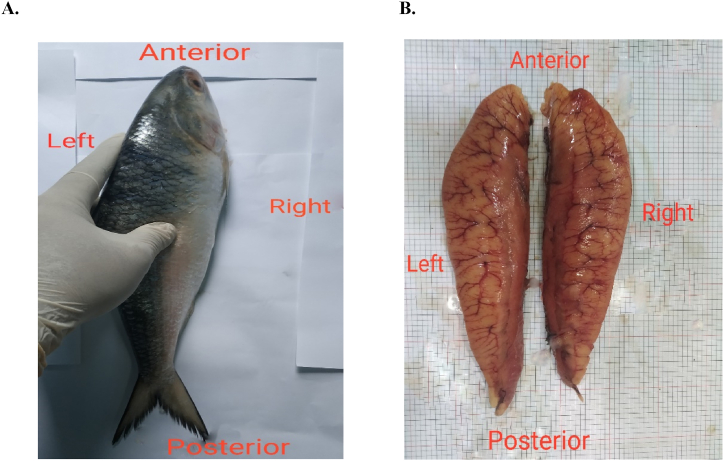
Directions and axes of Fish. A. Direction of fish body, B. Direction, and position of ovarian lobe.

**Fig. 3:**
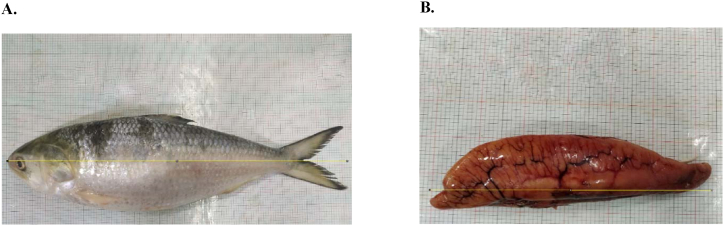
Measurement of fish and ovary A. measurement of body length, B. measurement of ovary.

The weight and body depth of the samples were measured with digital balance and slide calipers and each of them were placed under the digital camera to capture photographs. The samples were dissected from posterior to anterior part of their body and the gonads were collected with highest care (Fig. 2A). After wiping out the blood and dust, the photographs of gonads as well as left and right lobes of each gonad were taken placing them on a lined white graph paper (Fig. 2B). The images were then analyzed with *image j* (version 1.52 u)*,* and length of fish, gonad and each ovarian lobe were measured (Fig. 3A and B) [20].

### 2.3 Methods to determine peak breeding season

Two approaches were used in this study: i) Observing monthly variation in Gonado-Somatic Index (GSI) and ii) Observing gonadal development stage. Gonado-Somatic Index was calculated as GSI = (Gonad Weight/Total Body) *100% [21,22].

### 2.4 Determination of first maturity

Maturity stages of gravid Hilsa were determined by observing the egg size and characteristics as described by Ref. [23](Supplementary Table 1). Probit and Logit analysis was used to determine the size at maturity (M_50_) [22,24,25].

#### 2.4.1 Determining maturity stage

Gross physical (morphological) examination of the ovary was used to track the gonads' seasonal progression (stages of maturation) as described by Ref. [23] (Supplementary Table 1). The final stage-6 of the eggs was considered as ‘mature’ in our maturity study.

### 2.5 Counting of eggs

**Fig. 4:**
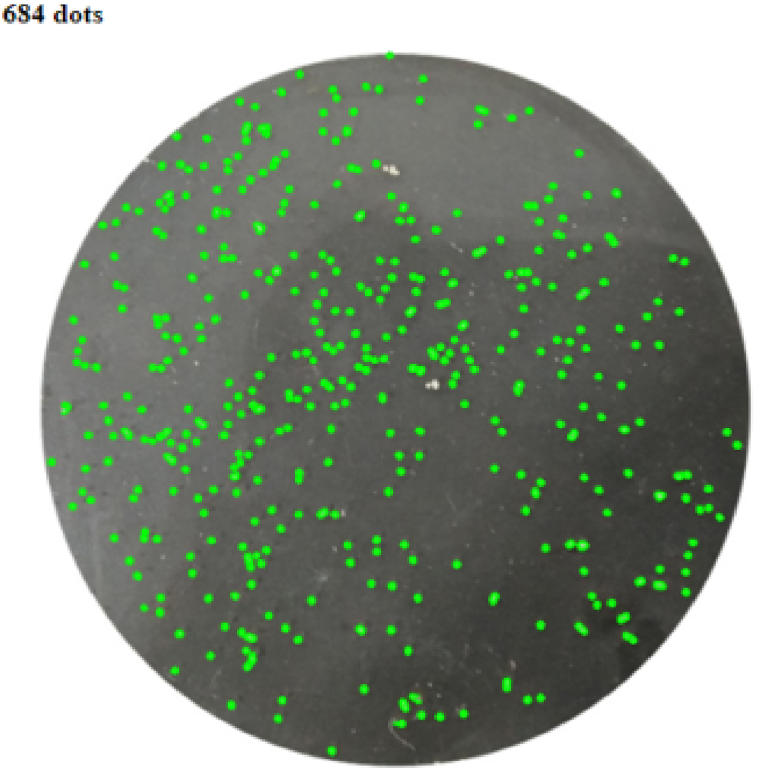
Counting eggs through ‘Dot count’ web portal.

The eggs of Hilsa were counted following a procedure developed in our lab as described in Ref. [26]. We used a combination of manual and software-based ‘dot counter’. The dot counter estimates the number of total eggs from a proceed image of Petri dish containing the eggs. We tried to process high quality images following their egg boiling method. To conduct the research, a certain number of eggs were taken from each of the front, middle and rare parts of each individual ovarian lobe of a fish as subsamples and boiled for 10 min using electric kettle keeping them in vacuumed polythene bags. After wiping out the water with paper towel, the subsamples were kept in open air for vaporization. Eggs were then placed in petri dishes with water and each single piece of eggs were separated using cotton bud. Finally, photographs were captured and utilized in the web-based platform (https://dotcounter.boopis.com) for counting eggs (Fig. 4).

### 2.6 Estimating fecundity

We used gravimetric method for estimating fecundity of Hilsa. Samples were taken from the front, middle and rare (back) parts of left and right lobes. The number of eggs found using the dot counter was considered as the count of each subsample and assigned as F1, F2, F3, F4, F5 and F6 accordingly. Weight of gonad was calculated and multiplied with the total average count of Average fecundity of the subsamples were calculated as: F_1_+ F_2_+ F_3_+ F_4_+F_5_+F_6_/6; and the final fecundity of the sample (F) was estimated as: average fecundity × total gonad weight.

### 2.7 Within and between-lobe variation in fecundity

For estimating variation in fecundity depending on sizes, fecundity was placed against total length and weight of fishes. Certain amount (0.05 gm) of eggs were taken from a part of the lobes (Front, Middle and Rare) to visualize the variation in egg count in two different lobes of an ovary and different regions of a lobe. The count of eggs was compared between two different lobes of an ovary and different parts of each lobe to figure out if any significant difference exists among the various regions of gonad regarding number of eggs.

### 2.8 Egg size estimation

**Fig. 5:**
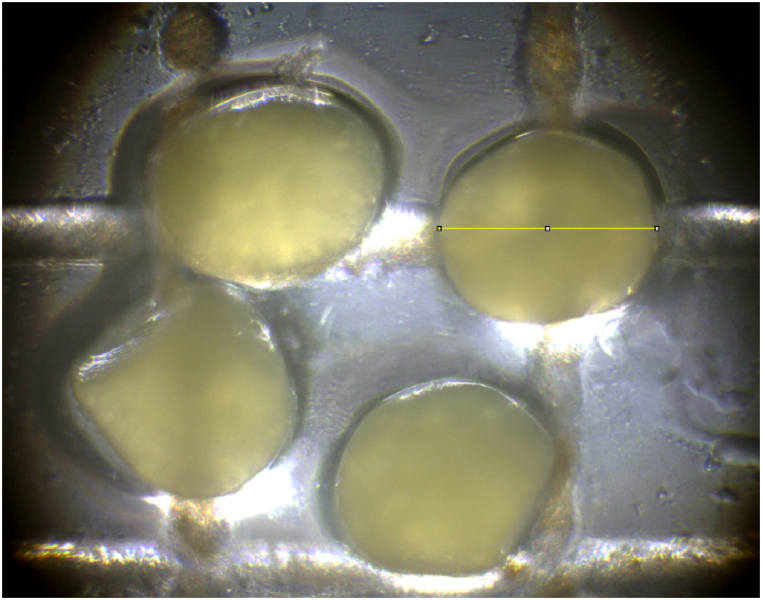
Egg size estimation using microscopic image taken by video-operated microscope camera.

To calculate egg size of Hilsa, eggs were placed under microscopic camera and photographs were taken to capture microscopic image of the eggs. Egg counting plate was used that was marked with a scale in millimeters. The images were then analyzed with image j (version 1.52u) software and diameter of the eggs were measured (Fig. 5). Furthermore, four or five individual eggs were taken from different regions of two different lobes of each fish to measure and the average diameter of those eggs was considered as the final size of the sample.

### 2.9 Within and between-lobe variation in egg size

Size of eggs (in micrometer) was placed against length and weight of fishes with a view to perceiving variation in size of eggs based on size of fishes. Twenty pieces of eggs were taken individually from the front, middle and rare parts of each ovarian lobe of a fish. The average size of the three regions of a lobe was considered as the final size of a single lobe whereas the average of the twenty individual eggs was considered as the final size of that region. Egg sizes were compared between two different lobes of an ovary and among different regions of a lobe to figure out if any significant difference exists.

### 2.10 Statistical analysis

Statistical analysis were conducted with MINITAB, Version 17 [27]. Tests for normality of the data were done according to Ref. [28]. To test the weight and fecundity variation between two lobes, a paired *t*-test was used, and to test the variation in egg size and fecundity among three parts of the lobe, a repeated measure ANOVA were used in generalized linear model (GLM). The individual fish ID, lobe and parts of the lobe were set in the model as factors. For multiple comparisons among the means, Tukey's HSD test was used for post-hoc analysis [29].

**2.11 Ethical approval:** The study was approved by the Animal Ethics Committee of Khulna University (approval # KRAEC-2020/0/06:July 28, 2020. The Animal Ethics Committee includes Professor AK Fazlul Hoque - Chairman (Professor Dr. Md. Nazmul Ahsan - member and Professor Dr. Sarder Safiqul Islam– member).

## 3 Results

### 3.1 Peak breeding season

**Fig. 6:**
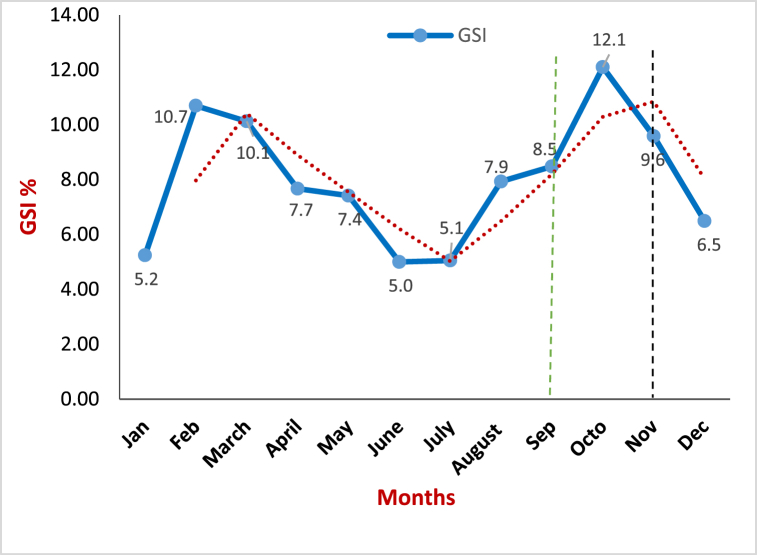
Seasonal variation of Hilsa's GSI value (% of body weight). Data are shown as the means of GSI. The dashed line represents the overall trend of the data, and the solid line represents the average GSI for the month.

The monthly variation in gonadosomatic index (GSI) of Hilsa (*Tenualosa ilisha*) is presented in Fig. 6. The highest GSI value was found in October (12.1), which suggests that Hilsa's breeding season is very close, which indicates that they may breed either within October or at the early days of November. From the end of November to January, the GSI values decreased gradually, but in February–March, a second peak of GSI (10.7–10.1) was observed. The GSI was remarkably lower from April to August (7.7–7.9).

### 3.2 Size at maturity

**Fig. 7:**
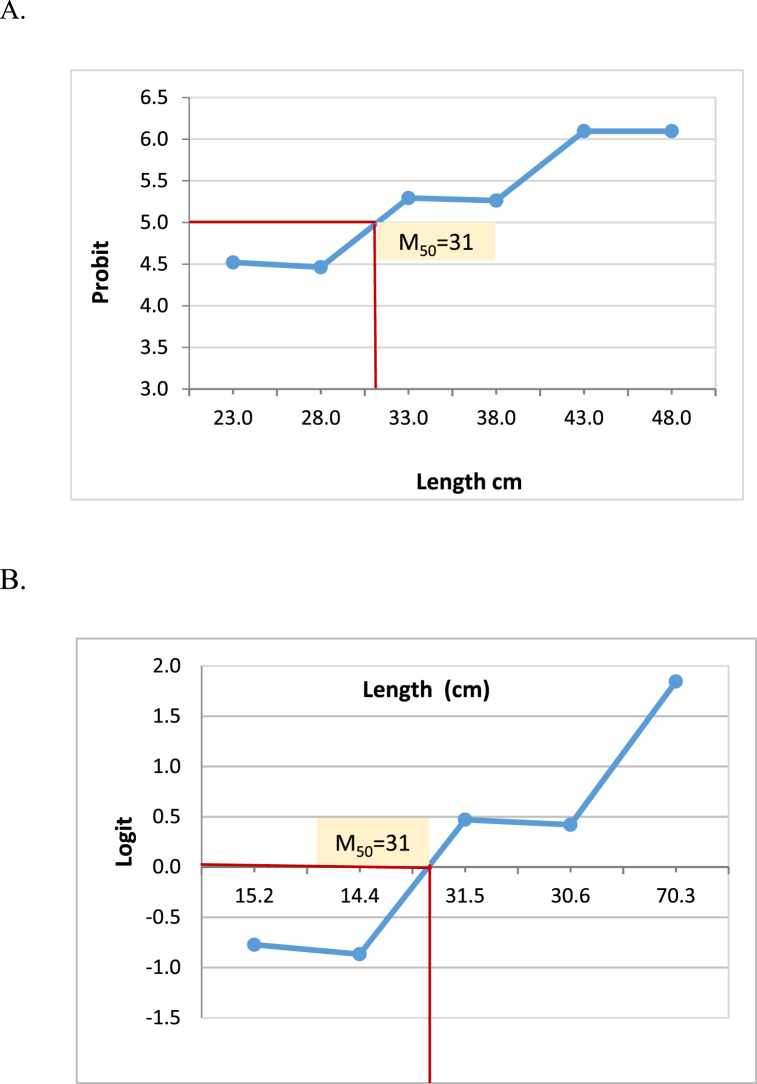
Probit and Logit values of mature female against the length of the fish (cm). A. Probit plot for M_50_. B. Logit plot for M_50_.

**Fig. 8:**
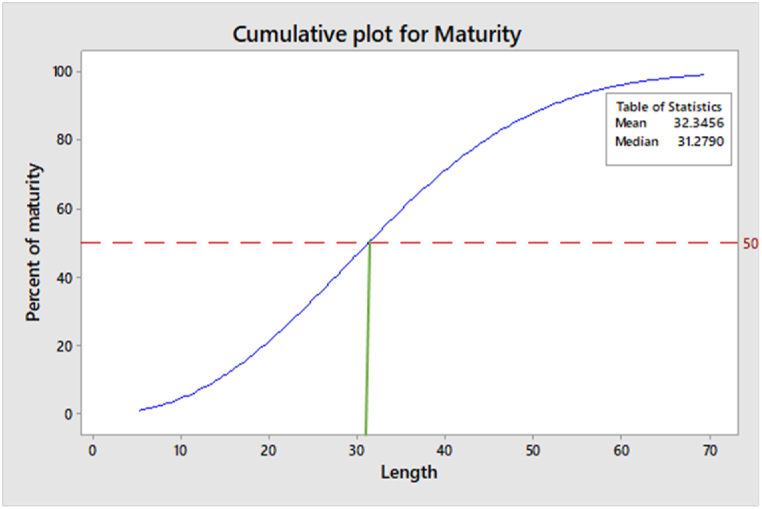
Cumulative probability plot of mature female Hilsa against total length (cm). The 50-50 chance of maturity is at 31.2 cm total length.

Probit analysis, Logit analysis and cumulative probability analysis estimated the maturity size of the *Tenualosa ilisha* (M_50_) as 31 cm (total length) (Fig. 7 A-B and 8). The 95% confidence interval of Maturity_50_ was measured as 28.8–33 cm total length through the cumulative plot for maturity (Fig. 8).

### 3.3 Variation in egg size

#### 3.3.1 Descriptive statistics of the samples

The descriptive statistics of lengths, body weights and lobe weights of the samples are given in Table 2. The average lengths, body size of the fish and weights of the lobes were recorded as 36.2 ± 3.7 cm, 640 ± 219g and 31.01 ± 13.7g respectively.

**Table 2:** Descriptive statistics of size and lobe weights of the specimen.

Sample	Length (cm)	Body weight (g)	Lobe weights (g)	
			Left lobe	Right lobe
1	43.16	1105	35	35
2	35.09	537	17	18
3	34.84	510	18	20
4	41.86	902	36	32
5	44.09	968	47	47
6	41.73	998	41	45
7	33.25	435	8	8
8	38.28	730	17	13
9	35.06	530	42	44
10	36.75	582	36	34
11	39.65	969	60	55
12	37.59	812	49	46
13	32.95	358	13	16
14	36.81	847	53	61
15	36.44	556	29	26
16	36.33	686	31	39
17	37.45	563	36	32
18	35.50	703	50	45
19	32.44	451	21	20
20	33.56	503	21	18
21	31.98	508	33	32
22	35.84	538	25	23
23	31.26	491	22	24
24	31.14	316	19	18
25	31.09	412	22	19
Average (mean ± std)	36.2 ± 3.7	640 ± 219	31.2 ± 13.8	30.8 ± 14
			31.01 ± 13.7	

#### 3.3.2 Size variation between the lobes

**Fig. 9:**
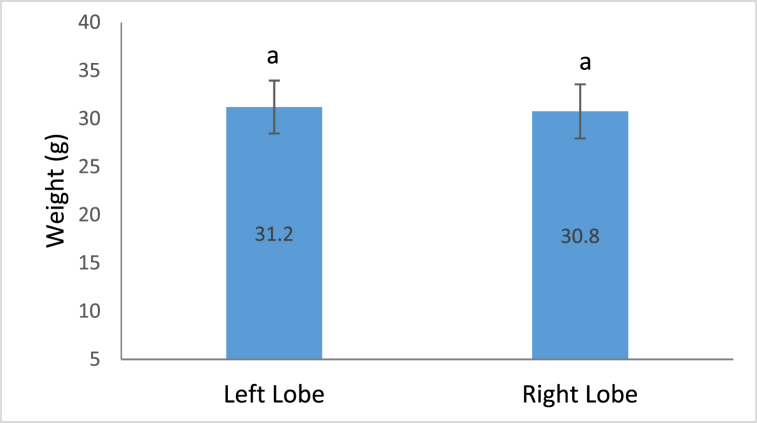
Variation in weights between left and right lobe. Mean weight of left lobe and right lobes. The data are presented as mean ± se.

No significant variation was observed for sizes between two lobes of a fish (t_(24)_ = 0.63, p = 0.53) (Fig. 9).

#### 3.3.3 Relationship between egg-size and size of fish

**Fig. 10:**
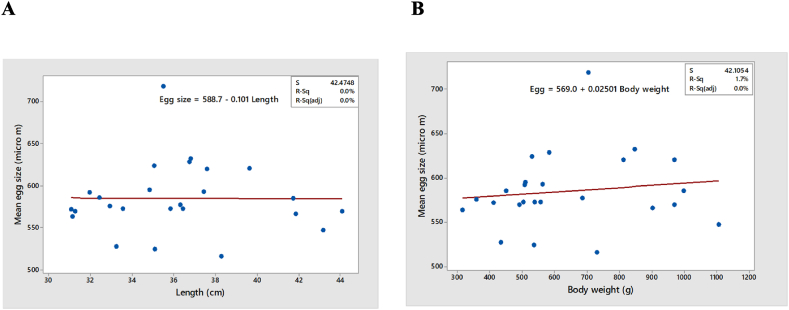
Relationship between size of egg and size of the fish. A. Relationship between egg size and length B. Relationship between egg size and body weight of fish.

The egg sizes did not vary significantly with the sizes of the fish, as no significant correlation was observed between the egg size andlength or body weight of the fish (Pearson correlation r of mean egg-size vs. length = −0.009, p = 0.966; and r = 0.132 and p = 0.530 for egg size vs. body weight) (Fig. 10A and B).

### 3.4 Relationship between total fecundity and fish size

**Fig. 11:**
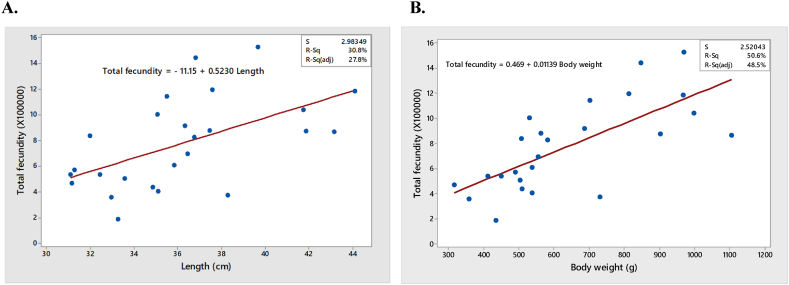
Relationship of fecundity with size of fish. A. Fecundity vs Length B. Fecundity vs Total body weight of fish.

The samples of gravid females ranged from 31.09 to 44.09 cm in total length, 316–1105 g in body weight and 0.186 to 1.493 million in fecundity (Table 2, Table 3). Total fecundity revealed strong correlation with length and weight of Hilsa (correlation co-efficient *r* of total fecundity and Body weight r = 0.7 and p = 0.000 for total fecundity vs. body weight; r = 0.6 and p = 0.004 for of total fecundity vs. length) (Fig. 11 A and B).

**Table 3:** Total and two lobes fecundity of the specimen.

Sample	Left lobe (in million)	Right lobe	Total fecundity
1	0.402	0.42	0.822
2	0.186	0.197	0.383
3	0.2	0.214	0.413
4	0.437	0.393	0.829
5	0.562	0.56	1.122
6	0.462	0.525	0.987
7	0.093	0.084	0.177
8	0.21	0.143	0.352
9	0.434	0.517	0.951
10	0.401	0.383	0.784
11	0.772	0.704	1.476
12	0.596	0.56	1.156
13	0.154	0.19	0.345
14	0.647	0.747	1.394
15	0.357	0.314	0.671
16	0.391	0.495	0.886
17	0.447	0.402	0.849
18	0.582	0.521	1.103
19	0.267	0.252	0.519
20	0.263	0.226	0.489
21	0.41	0.4	0.81
22	0.309	0.28	0.588
23	0.263	0.288	0.55
24	0.241	0.213	0.454
25	0.282	0.238	0.52
Average (mean ± std)	0.375 ± 0.16	0.371 ± 0.17	0.745 ± 0.33
	0.373 ± 0.17

#### 3.4.1 Relationship between fecundity/kg body weight and size of fish

**Fig. 12:**
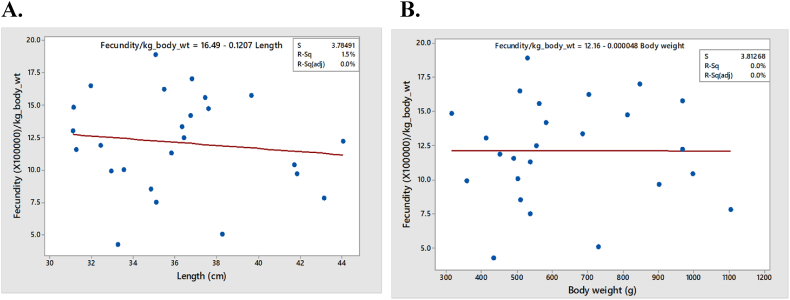
Relationship between fecundity/kg body weight and size of fish. A. fecundity/kg body weight vs Length B. fecundity/kg body weight vs Total body weight of fish.

Fecundity per kg body weight indicates relative fecundity of fishes according to sizes. The two figures below show that there is no correlation among fecundity/kg body weight vs length (P > 0.05) and fecundity/kg body weight vs body weight (P > 0.05) of fishes (Fig. 12A and B).

### 3.5 Between-lobe and within-lobe variation in egg size

**Fig. 13:**
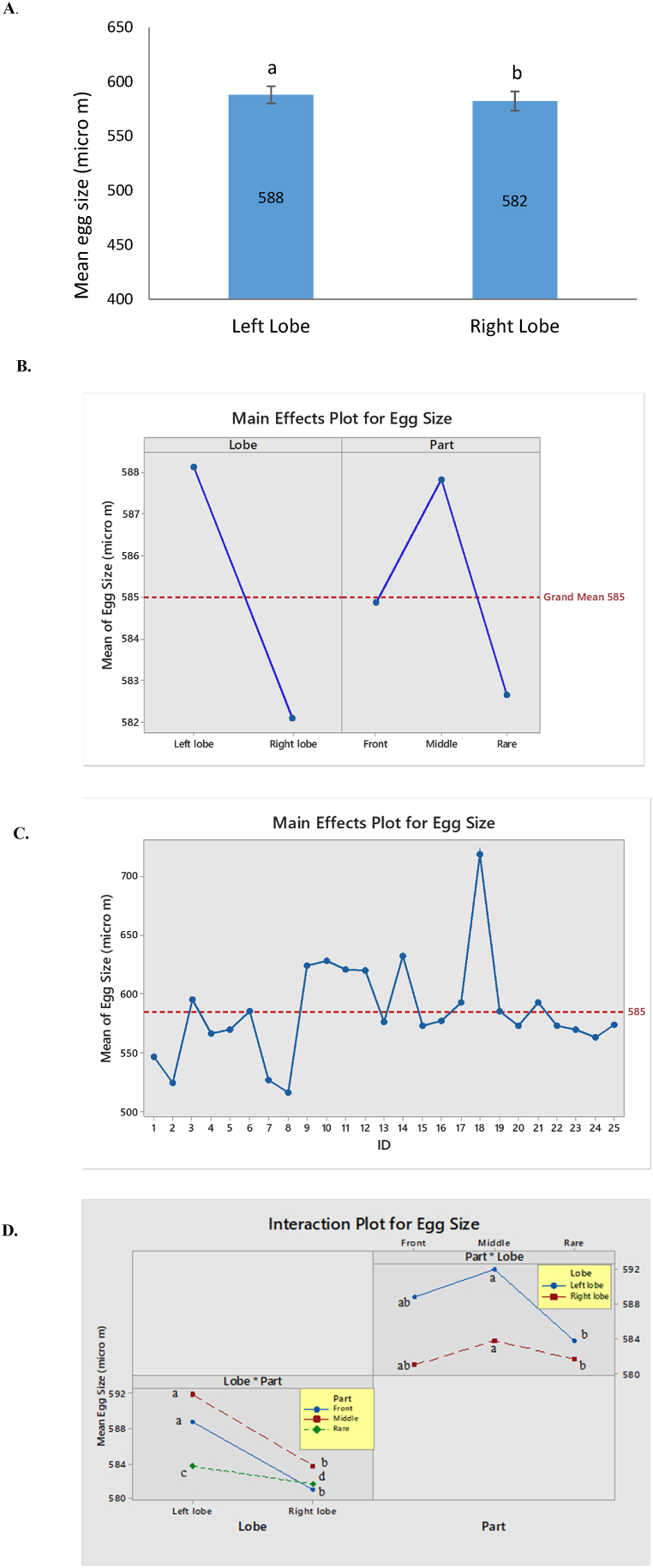
Egg size: Intra and inter lobe variation in egg size of Hilsa (*T. ilisha*). A. Mean difference in egg size between left and right lobe. B. Main effects plot for egg size in different lobes and parts of the lobes C. Main effects plot for egg size in different individuals. D. Interaction plot for egg-sze between lobes and parts of lobe. The figure that does not share any common letter (a, b, c) is significantly different at p < 0.05.

A significant variation was observed in egg sizes between the left and right lobes of the fish (*t*_(24)_ = 2.42, p = 0.024) (Fig. 13 A). It is evident form the figure that egg size varied significantly between left lobe and right lobe; and among the parts of the lobes of Hilsa (*F*_(24,122)_ = 110.83, p = 0.00 for fish ID; *F*_(1,122)_ = 14.57, p = 0.00 for lobe and *F*_(2,122)_ = 3.6, p = 0.03 for parts of lobe).

(Fig. 13B–D). Fig. 13D presents interactions among the variables that show pairwise comparison between left and right lobe of a fish and three different parts of a lobe. Egg size varied significantly between left and right lobe and three parts of a lobe (P < 0.05).

### 3.6 Between-lobe and within-lobe variation in fecundity

**Fig. 14:**
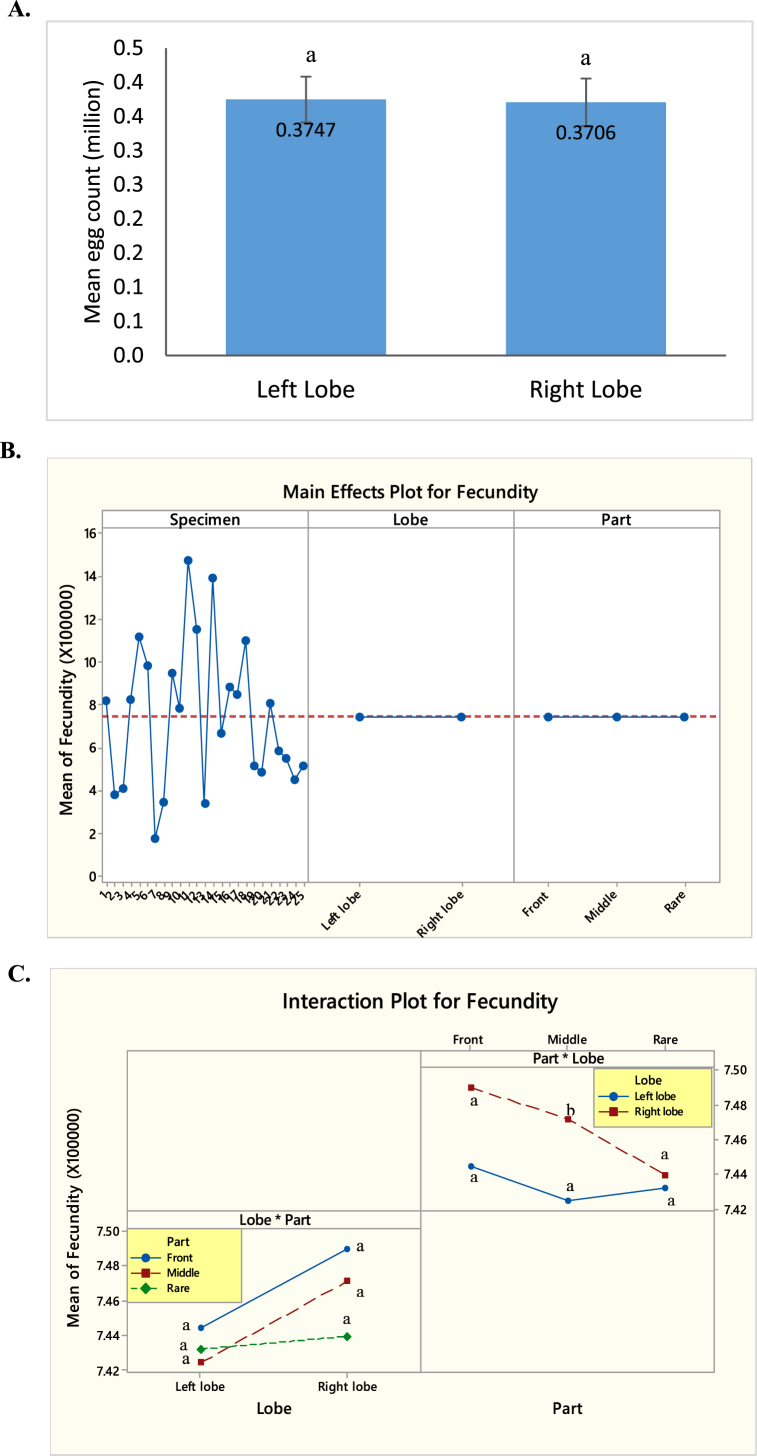
Variation in fecundity between left and right lobe and three parts of each lobe. A. Mean egg count between left and right lobe. B. Main effects plot for fecundity based on lobes, parts of lobe and specimen. C. Interaction plot for fecundity based on lobes and parts of lobe.

No significant variation was observed in egg count between the left and right lobes of the fish (t _*(24)*_ = 0.41, p = 0.687) (Fig. 14 A). It is evident form the figure that fecundity did not vary significantly between left lobe and right lobe; and among the parts of the lobes of Hilsa but varied among the individual specimen (F_*(24,122)*_ = 1181.77, p = 0.00 for fish ID; F_*(1,122*_) = 0.72, p = 0.398 for lobe and F_*(2,122)*_ = 0.22, p = 0.805 for parts of lobe) (Fig. 14 B–C). Fig. 13D presents interactions among the variables that show pairwise comparison between left and right lobe of a fish and three different parts of a lobe. Egg size did not vary significantly between the left and right lobe and three parts of a lobe.

## 4 Discussion

### 4.1 Peak breeding season

We were able to identify two distinct breeding peaks: the highest one in October, and the second one in February–March (Fig. 6). However, the first peak of October was very clear and superseded all the GSI values, which indicates the peak breeding season of Hilsa. Whereas the second peak in February–March was relatively mild. Several studies with Hilsa (*Tenualosa ilisha*) reported two peaks of breeding in Bangladesh. Quddus et al. [30] report two breeding seasons of Hilsa; one in between July–October and another in January–March. Besides, Hossain [31] observes the spawning season of Hilsa shad as June–November and January–April. Observing two breeding peaks is not surprising because there are many other aquatic species are reported to have two breeding peaks [22,32,33,34]. In several recent studies, peak breeding season of Hilsa shad has been found around October [7],[35]. Flura et al. [35]and Rahman et al. [7] observed the peak breeding season of Hilsa in Bangladesh as October and September–October respectively. Our study shows the results from the most recent data of Hilsa, which determines the breeding season precisely. Previous works are also in corroboration with the findings of the present results.

### 4.2 Size at maturity

Usually the mean size at first maturity is chosen as the minimum legal size. In Hilsa, the minimum legal size has typically been used for the previous few years to be 50% maturity. In the current study, female Hilsa's size at first maturity was determined to be 31 cm total length. Amin et al. [36] recommends that the minimum size of capture of Hilsa shad (*Tenualosa Ilisha*) in Bangladesh should be 28–30 cm total length. Bangladesh government, based the instructions from department of fisheries, is now implementing 25 cm (total length) as a legal size of Hilsa, which is smaller than the result of our study [37]. suggests that the size at first maturity of female Hilsa should be 28–30 cm [38]. observed maturity size as 31–33 cm, whereas [39] found it as 30–35 cm. In our neighboring country India [40], estimated this size as 20.7–20.8 cm. However, several studies were conducted in the Middle East countries like Iraq, Iran, where they report the maturity size as 33 cm [41,42]. This variation might be attributed to different geographical location, water quality and some other environmental factors.

We investigated a total of 391 specimen of Hilsa from seven different regions in Bangladesh over the last year and found mean size at first maturity (M50) of Hilsa as 31 cm total length. Therefore, this result is reliable and can be applied as the source of further actions for Hilsa fishery management and conservation policy in Bangladesh. We suggest that the existing legal size of Hilsa should be revised, because the present practice of 25 cm legal size of capture was determined based on scape size of gravid females through the net of a particular mesh sizes. It was not an appropriate scientific method for estimating size at first maturity (50-50 chance of being mature or immature). However, in our study, the mean size at maturity was determined only for female. So, in order to set up a legal capture size for Hilsa across Bangladesh, further research on sexual maturity of male Hilsa is recommended.

### 4.3 Variation in egg size

The study quantitatively evaluated variation in egg size in two different lobes of an ovary; and at three parts of a lobe (front, middle and rare). Consistent with expectation, we found substantial differences in egg size between left and right lobe. Besides, egg size significantly varied among front, middle and rare parts of each lobe. Eggs from middle part of the lobe showed bigger size than the front and rare part. Maybe, the structure of the ovary is the reason behind it. However, we found no significant relationship between egg size and body length and between egg size and fish body weight. We noticed that some big fish produced small eggs; and some small fish produced large eggs. This result is not unusual, because the size of the egg depends on the maturity stage of the fish, regardless of body size. Measuring three thousand of eggs, we observed that size of egg varied between 0.413 and 810.21 μm in diameter and mean value of size is 582.54 μm. The outcome of this study agrees with some other studies [43,44]. Haldar et al. [44] estimated egg size of Hilsa in Bangladesh as 0.7–0.9 μm. Saifullah et al. [43] reports eggs size of Hilsa as 0.66–0.88 μm, whereas Islam et al. [45] found the egg size varied from 0.3 to 0.6 μm. Mathur [46] examined egg size of Hilsa from Hugly river in West Bengal of India as 0.76–0.86 μm.

### 4.4 Variation in fecundity

In our present study the fecundity of eggs ranged from 0.186 million to 0.1493 million. The fish with 39.65 cm length and 969 g body weight was found to carry the largest highest number of eggs (total egg weight 115 g). The fecundity of fish with similar length and weight, however, varied. A fish measuring 35.09 cm in total length, 537 g in body weight and 37 g in gonad weight produced 0.365 million eggs, whereas another fish of the same length and body weight with 85 g of egg weight produced 0.185 million eggs. Fish exhibit a wide range in fecundity, and an individual's egg production depends on a variety of variables, including size, age, physical condition, and others. Mookerjee and Mazumdar [47] concluded that reproductive capacity of a fish varies with space availability and food supply. This type of variation was also documented by several studies [43,48,49]. Mondal et al. [48] reports fecundity of Hilsa from Patuakhali of Bangladesh as 0.169 million to 1.09 million. Mazid [50] estimated the fecundity ranging from 0.095 to 0.112 million. Akter et al. [49] found maximum fecundity of 1.239 million in Padma River, Rajshahi [43]. reports fecundity of Hilsa ranging from 1 to 1.9 million.

Analysis of fecundity showed that the relationship between fecundity and total length and body weight were found significant (p < 0.05). However, there was no significant relationship between fecundity/kg body weight and size of fish. In addition, counting eggs from different parts of the lobe has no relationship among them. Therefore, we can infer that taking samples from three different regions of an ovarian lobe is necessary for fecundity study of Hilsa.

## 5 Conclusion

Our study confirms that the peak breeding season of Hilsa (*Tenualosa ilisha*) is October. The government of Bangladesh is currently prohibiting the capture of berried Hilsa from October to November. Considering the results of our investigation, we would advise that October might be the appropriate time to impose a ban on catching Hilsa fish. The present study proves that female Hilsa from different populations in Bangladesh attain maturity (M_50_) at 31 cm total length, which suggests the revision of the existing minimum legal capture size of 24 cm.

Our study also shows that egg size varies significantly between two lobes of an individual and between three parts of each lobe. Eggs from the middle part of each lobe are bigger in size than the other two parts (front and rare). However, egg size has no relationship with length and size of Hilsa, rather it was dependent on the maturity stage of the eggs. Total fecundity varied significantly with total length and body weight of Hilsa but did not vary between two lobes and between three different parts of each lobe. The study suggests that samples from three different parts of gonad is not mandatory in fecundity count, as fecundity did not vary significantly between the lobes and parts of lobe. However, for making any inferences on egg-size, we should take the parts of the lobes into consideration.

The findings of our study would definitely assist in proper conservation and management of Hilsa in general, and increase the insight into the study of reproductive biology in particular.

## Author contribution statement

Md. Sayeed Abu Rayhan: Conceived and designed the experiments; Performed the experiments; Analyzed and interpreted the data; Wrote the paper.

Md. Shohanur Rahman: Performed the experiments; Contributed reagents, materials, analysis tools or data.

Protick Kumar Bose; Md. Golam Sarower: Contributed reagents, materials, analysis tools or data.

Muhammad Yousuf Ali: Conceived and designed the experiments; Analyzed and interpreted the data; Wrote the paper.

## Data availability statement

Data included in article/supplementary material/referenced in article.

## Additional information

No additional information is available for this paper.

## Declaration of competing interest

The authors declare that they have no known competing financial interests or personal relationships that could have appeared to influence the work reported in this paper.

## References

[1] Hossain M.A., Almatar S.M., Al-Hazza A.A. (2014). Proximate, fatty acid and mineral composition of hilsa, Tenualosa ilisha (Hamilton 1822) from the Bay of Bengal and Arabian Gulf. Indian J. Fish..

[2] Ganguly S., Mahanty A., Mitra T., Mohanty S., Das B.K., Mohanty B.P. (2018). Nutrigenomic studies on hilsa to evaluate flesh quality attributes and genes associated with fatty acid metabolism from the rivers Hooghly and Padma. Food Res. Int..

[3] Rahman M.J., Wahab M.A., Amin S.N., Nahiduzzaman M., Romano N. (2018). Catch trend and stock assessment of Hilsa Tenualosa ilisha using digital image measured length‐frequency data. Marine and Coastal Fisheries.

[4] Wahab M.A., Beveridge M.C.M., Phillips M.J. (2019).

[5] Hossain M.S., Sharifuzzaman S.M., Rouf M.A., Pomeroy R.S., Hossain M.D., Chowdhury S.R., AftabUddin S. (2019). Tropical hilsa shad (T*enualosa ilisha*): biology, fishery and management. Fish Fish..

[6] Rahman M.J., Wahab M.A., Nahiduzzaman M., Haque A.B.M.M., Cohen P. (2020). IOP Conference Series: Earth and Environmental Science.

[7] Rahman M., Pramanik M., Flura A.T., Hasan M. (2017). Impact assessment of twenty-two days fishing ban in the major spawning grounds of *Tenualosa ilisha* (Hamilton, 1822) on its spawning success in Bangladesh. J Aquac Res Develop.

[8] Rahman M.A., Ahmed T., Pramanik M.M.H., Alam M.A. (2015). Impact of fifteen days fishing ban in the major spawning grounds of hilsa (*Tenualosa ilisha* Hamilton 1822) on its spawning success. Research in Agriculture Livestock and Fisheries.

[9] (2019). Prothom Alo.

[10] DoF (2017).

[11] Miah M.S. (2015). Climatic and anthropogenic factors changing spawning pattern and production zone of Hilsa fishery in the Bay of Bengal. Weather Clim. Extrem..

[12] Hossain M.S., Sharifuzzaman S.M., Chowdhury S.R., Sarker S. (2016). Habitats across the life cycle of hilsa shad (*Tenualosa ilisha*) in aquatic ecosystem of Bangladesh. Fish. Manag. Ecol..

[13] Hossain M.S. (2017). The shifting habitat of. Hilsa: River to Sea.

[14] Giri S., Chanda A., Maity S., Chakraborty K., Hazra S. (2022). Role of tide and lunar phases on the migration pattern of juvenile Hilsa shad (*Tenualosa ilisha*) within a meso‐macrotidal estuary. J. Fish. Biol..

[15] Rahman M.A., Ahmed T., Pramanik M.M.H., Flura H.M., Hasan M., Riar G.S., Mahmud Y. (2017). On-board breeding trial of hilsa (Tenualosa ilisha, Ham. 1822) and testing of larval rearing in Bangladesh. J. Aquacult. Res. Dev..

[16] Chattopadhyay D.N., Chakraborty A., Ray P.K., Jayasankar P. (2019). Preliminary observations on artificial fecundation, hatching and developmental stages of embryo and larvae of the Indian shad Tenualosa ilisha (Hamilton, 1822). Indian J. Fish..

[17] Chattopadhyay D.N., Chakraborty A., Ray P.K., Mandal R.N., Das A. (2021). Transportation of fertilized eggs and yolk-sac larvae of hilsa shad, Tenualosa ilisha (Hamilton, 1822) in different transportation systems. Aquaculture.

[18] Chattopadhyay D.N., Chakraborty A., Ray P.K., Mandal R.N., Suresh V.R., Banik S.K. (2018). First ever weaning and feeding behavior of Hilsa Shad, Tenualosa ilisha (Hamilton, 1822) fry under captive culture in freshwater pond. Environ. Ecol..

[19] Chattopadhyay D., Chakraborty A., Ray P.K., Mandal R., Banik S.K., Suresh V.R., Ghosh K. (2019). Larval rearing of hilsa shad, Tenualosa ilisha (Hamilton 1822). Aquacult. Res..

[20] Schneider C.A., Rasband W.S., Eliceiri K.W. (2012). NIH Image to ImageJ: 25 years of image analysis. Nat. Methods.

[21] Alam A., Gopinath V., Kumar J., Das S.C.S., Jha D.N., Joshi K.D., Srivastava R.S. (2020).

[22] Ali M.Y., Hossain M.B., Sana S., Rouf M.A., Yasmin S., Sarower M.G. (2020). Identifying peak breeding season and estimating size at first maturity of mud crab (Scylla olivacea) from a coastal region of Bangladesh. Heliyon.

[23] Pillay T. (1973). The role of aquaculture in fishery development and management. JFisheries Board of Canada.

[24] Campbell G.R. (1986). Proceedings of the.

[25] Overton J.L., Macintosh D.J. (2002). Estimated size at sexual maturity for female mud crabs (genus Scylla) from two sympatric species within ban don bay, Thailand. J. Crustac Biol..

[26] Raqib (2019).

[27] Minitab I. (2014).

[28] Anderson T.W., Darling D.A. (1954). A test of goodness of fit. J. Am. Stat. Assoc..

[29] Tukey J.W. (1949). Comparing individual means in the analysis of variance. Biometrics.

[30] Quddus M., Shimizu M., Nose Y. (1984).

[31] Hossain M.M. (1985).

[32] Prasad P., Sudarshana R., Neelakantan B. (1988). Feeding ecology of the mud crab, *Scylla serrata* (Forskal) from Sunkeri back waters, Karwar. J. Bombay Nat. Hist. Soc..

[33] Rasheed S., Mustaquim J. (2010). Size at sexual maturity, breeding season and fecundity of three-spot swimming crab Portunus sanguinolentus. (Herbst, 1783)(Decapoda, Brachyura, Portunidae) occurring in the coastal waters of Karachi.

[34] Fahimi N., Seyfabadi J., Sari A. (2017). Size at sexual maturity, breeding season, and fecundity of the intertidal xanthid crab Leptodius exaratus (H. Milne Edwards, 1834)(Decapoda: Brachyura) in the Persian Gulf, Iran. J. Crustac Biol..

[35] Flura M.Z., Rahman B.S., Rahman M.A., Ashraful M., Alam M., Pramanik M.H. (2015). Length-weight relationship and GSI of hilsa, *Tenualosa ilisha* (Hamilton, 1822) fishes in Meghna river, Bangladesh. Int J Nat Soc Sci.

[36] Amin S.N., Arshad A., Haldar G.C., Shohaimi S., Ara R. (2005). Estimation of size frequency distribution, sex ratio and length-weight relationship of Hilsa (*Tenualosa ilisha*) in the Bangladesh water. Res. J. Agric. Biol. Sci..

[37] Pillay S.R. (1964).

[38] Islam M.S., Huq O.M., Hossain M., Asad S.A., Das N.N. (1989). Hilsa Investigations in Bangladesh, Marine Fishery Resources Management.

[39] Dunn I.G. (1982). The Hilsa fishery of Bangladesh, 1982: an investigation of its present status with an evaluation of current data. A report prepared for the Fisheries Advisory Service, Planning, Processing and Appraisal Project. Field document.

[40] Bhaumik U., Mukhopadhyay M.K., Shrivastava N.P., Sharma A.P. (2012). The largest recorded Hilsa (*Tenualosa ilisha*) in India from Tapti estuary, Gujarat. Fish. Chimes.

[41] Blaber S.J.M., Mazid M.A. (2001). Hilsa fishery research in Bangladesh. ACIAR Proj.

[42] Hussain N.A., Jabir M.K., Yousif U.H. (1994). On the biology of sbour *Tenualosa ilisha* (Hamilton) in the Shatt Al-Arab River, south of Iraq, with notes on their distribution in the Arabian Gulf. Mar. Mesopotamica.

[43] Saifullah A.S.M., Rahman M.S., Khan Y.S.A. (2004). Fecundity of *Hilsa ilisha* (Hamilton, 1822) from the bay of bengal. Pak. J. Biol. Sci..

[44] Haldar G.C., Wahab M.A., Puvanendran V., Phillips M.J. (2012). Hilsa: Status of Fishery and Potential for Aquaculture, Proceedings of the Regional Workshop Held in Dhaka.

[45] Islam B.N., Talbot G.B. (1968). Fluvial migration, spawning, and fecundity of Indus River hilsa, *Hilsa ilisha*. Trans. Am. Fish. Soc..

[46] Mathur P.K. (1964). Studies on the maturity and fecundity of the hilsa, *Hilsa ilisha* (Ham.) in the upper stretches of the Ganga. Indian J. Fish..

[47] Mookerjee H.K., Mazumdar S.R. (1946). On the life history, breeding and rearing of Anabas testudineus (Bloch). J Dep Sci Cal Univ.

[48] Mondal B.K., Devnath S., Shaha D.C., Khan M.N.A., Choi J.-S. (2008). Relationships between fecundity and total length, body weight, ovary length, and ovary weight of hilsa shad, *Tenualosa ilisha* Hamilton, in Patuakhali, Bangladesh. Fish. Aquat. Sci..

[49] Akter M.A., Hossain M.D., Hossain M.K., Afza R., Bhuyian A.S. (2007). The fecundity of *Hilsa ilisha* from the river Padma near Godagari of Rajshahi district. Univ. J. Zool., Rajshahi Univ..

[50] Mazid M.A. (1998). Proceedings of BFRI/ACIAR.

